# Barriers and Facilitators for Participation in Global Health Research Training Programs Among Underrepresented Minority Groups

**DOI:** 10.4269/ajtmh.24-0847

**Published:** 2025-05-13

**Authors:** Usha Ramakrishnan, Monique Hennink, Kofi A. Kondwani, Radhika L. Sundararajan, Riley Hunt, Donna J. Ingles, Dionne Williams, Champagnae Smith, Lan Tran, Teris Taylor, Douglas C. Heimburger, Linnie M. Golightly

**Affiliations:** ^1^ Hubert Department of Global Health, Emory University, Atlanta, Georgia;; ^2^ Morehouse School of Medicine, Atlanta, Georgia;; ^3^ Weill Cornell Medicine, New York, New York;; ^4^ Vanderbilt University, Nashville, Tennessee;; ^5^ Vanderbilt University Medical Center, Nashville, Tennessee

## Abstract

The optimal global health (GH) workforce should be racially and ethnically diverse, yet few persons from historically underrepresented minority (URM) groups in the United States participate in GH training programs. We conducted a study to explore barriers and facilitators for URM individuals to participate in the NIH Fogarty International Center’s GH Program for Fellows and Scholars (FGHFS), which offers yearlong international research training opportunities. We used an exploratory sequential mixed methods study design that used qualitative in-depth interviews (*n* = 18) to inform a subsequent quantitative online survey (*n* = 82). We assessed URM interest and engagement in GH training at three stages of FGHFS (applicants, alumni, and eligible persons who had not applied). Most participants in both phases were female, Black or African American, aged between 31 and 39 years, and had completed graduate or postgraduate training; a third or less were Hispanic. We identified four principal barriers to participation in GH training programs including lack of exposure to GH, lack of mentorship or support, challenges of global travel and work, and finances. The barriers compounded across training stages. Principal facilitators of training engagement included encouraging mentors and supportive families. Recommendations for increasing the participation of URM individuals in GH research training programs included increased financial support and exposure to GH in academic studies, as well as exposure to role models and mentors who can provide career advising in GH. Our findings suggest that early exposure, mentorship, and sufficient financial support will facilitate URMs’ entry into GH.

## INTRODUCTION

Representation of historically underrepresented minorities (URMs) in global health (GH) services, initiatives, policies, and research is important to empower diverse perspectives, experiences, and contributions.^1^ However, few URM individuals participate in GH training programs that may lead them to careers in GH. The Enhancing Diversity for Global Equity Study aimed to understand the barriers and facilitators faced by URM individuals around participating in GH research training programs. This knowledge may be used to develop strategies to encourage or facilitate greater participation of URMs in GH training programs and careers.

URM is a term used to identify individuals from racial and ethnic groups that have been shown by census data and other federal measuring tools to be underrepresented on a national level. It also refers to groups who have been denied access and/or suffered past institutional discrimination in the United States. The NIH and National Science Foundation define URMs to include people who are Black or African American, Hispanic or Latino, American Indian or Alaska Native, Native Hawaiian or from other Pacific Islands.^2^^,^^3^ People from URM backgrounds are not well represented in higher education as a whole or in specific scientific fields such as GH. Less than half of the governmental public health workforce identifies as racial or ethnic minorities, yet most of the communities they serve are from URM backgrounds.^4^ For instance, only 6% of public health students identify as Hispanic American despite 12% of the United States population being Hispanic.^5^

Improving diversity in the public health workforce, and specifically within GH, is critical for effective service delivery to reduce health disparities and improve health outcomes.^6^^,^^7^ The U.S. Department of Health and Human Services advocated for more diversity in public and GH training to enhance health systems and boost minority participation in health-related services.^8^ Studies show that public health practitioners from URM backgrounds are better equipped to serve communities with the same race, ethnicity, culture, or language.^4^ The participation of URMs in GH roles has enormous benefits. It leads to more diverse voices in decision-making that can create better-informed, well-rounded GH services and interventions that benefit a broader range of individuals and communities.^1^

We conducted a mixed-methods study to understand barriers and facilitators encountered by people from URM backgrounds around participating in the NIH Fogarty International Center’s (FIC’s) GH Program for Fellows and Scholars (FGHFS).^9^ This 1-year training program supports early-career fellows and scholars from the United States and from low- and middle-income countries (LMICs) to design and conduct research projects in LMIC institutions with dual mentorship from United States and LMIC mentors. The postulate of FGHFS is that participants will become leaders in GH capable of catalyzing change through research to span the economic divide between high- and low-income countries. The immersive 1-year experience provides a foundation for scientific excellence, bilateral cultural appreciation, and awareness to permit collaborative international research. However, from its inception, the program has struggled to attract URM participants, which led to our interest in conducting the current study.

We examined interest and engagement in GH training at three different stages: 1) general interest in GH training, 2) applying to the FGHFS, and 3) participation in FGHFS. By focusing on when specific barriers occurred and how they operated at each stage, we aimed to provide a more detailed understanding and to develop targeted recommendations to increase URM participation in GH training programs.

## MATERIALS AND METHODS

We used an exploratory sequential mixed-method study design to assess the three stages of URM participation in GH programs. First, we conducted exploratory qualitative research (Phase 1) to identify and contextualize the barriers and facilitators experienced by URMs in considering or engaging in the Fogarty GH training program. Results of the qualitative interview phase were analyzed in depth and used to develop a quantitative survey (Phase 2) to measure the prevalence of the barriers and facilitators in a wider population of persons from URM backgrounds.

The study was a collaboration among Emory University, Vanderbilt University Medical Center, Weill Cornell Medicine, and Morehouse School of Medicine (MSM), supported by FIC (D43 TW009337). Data collection and analysis were conducted by Emory University and MSM with ethical approval from their institutional review boards. For the qualitative phase, the purpose, methods, and ethical considerations (e.g., protection of personal information) of the study were verbally explained to all participants before beginning each interview. Consent to participate in the study including allowing staff to record the interview was obtained and confirmed before each interview. Participants were informed they could withdraw their consent at any time. For the quantitative survey, electronic consent was obtained via Research Electronic Data Capture (REDCap).^10^^,^^11^ Details of the methods for the two sequential phases of the study are described below.

### Qualitative data collection (phase 1).

To be eligible for the study, participants needed to self-identify as a member of a URM group. We purposively recruited three groups of participants to capture the experiences of URMs with different types of engagement with GH programs. Group 1 (FGHFS alumni) included URMs who had completed a FGHFS fellowship. Group 2 (applicants) included URMs who applied to the FGHFS but were not accepted or withdrew their applications. Group 3 (potential applicants) included URMs who would be eligible for the FGHFS but have not applied. To recruit participants for Groups 1 and 2, we used the FGHFS database of applicants and databases of current and former fellows from six FGHFS consortia that were funded for the 10 years comprising 2012–2022 (Vanderbilt, Emory, Cornell, and Duke; University of North Carolina, Johns Hopkins, Morehouse, and Tulane; University of California, San Francisco, University of California, Los Angeles, University of California, San Diego, and University of California, Davis; Harvard, Boston University, Northwestern, and University of New Mexico; University of California, Berkeley, Yale, University of Arizona, and Stanford; University of Washington, University of Michigan, University of Hawaii, University of Minnesota, and Indiana University). From each database, we purposively selected individuals to provide diversity by URM classification, gender, URM ethnicity, and FGHFS program year. Selected individuals were invited to participate in the study via e-mail. To recruit participants for Group 3, we used advertisements and a snowball sampling strategy, because this group was harder to identify. Study announcements were distributed by all six FGHFS consortia, as well as at an annual meeting of the Consortium of Universities for GH (CUGH), through historically Black colleges and universities and URM-serving institutions that were not part of any existing consortia. Consortium members distributed the announcement to current fellows and alumni, with encouragement to participate.

Qualitative data were collected via in-depth interviews to enable a deeper and contextualized understanding of the participants’ experiences. We used three semi-structured interview guides, tailored to each subgroup of participants. Topics included: exposure to/interest in a GH career (all groups), application to FGHFS (groups 1 and 2), participation in FGHFS (group 1), and recommendations to increase URM participation in GH training (all groups). Interviews lasting 30–60 minutes were conducted and recorded using Zoom. Video cameras were on during the interviews to facilitate rapport building and more effective probing. Interviews were conducted by a trained PhD-level public health professional (Dionne Williams).

**Figure 1.**
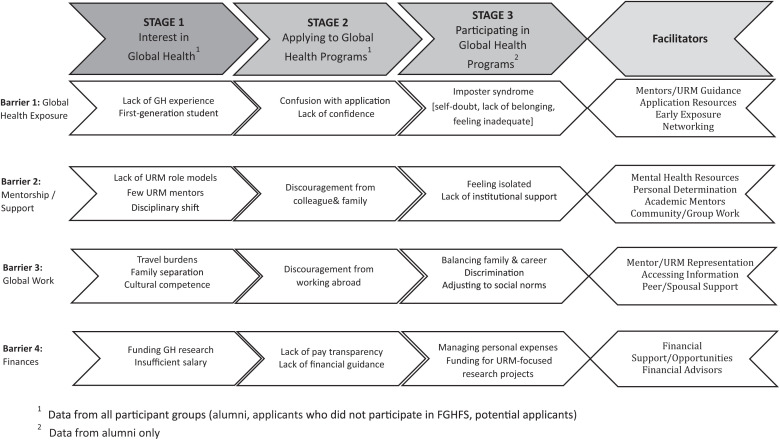
Barriers and facilitators for underrepresented minority (URM) participation in global health programs, by training stage. This framework illustrates ways in which each of the four principal barriers to pursuit of global health training is present at various stages of the participants’ training, as well as facilitators relevant to each barrier.

Interview transcripts generated using Zoom’s transcription function were reviewed and edited for accuracy by two separate analysts. All transcripts were deidentified and uploaded into MAXQDA^12^ for data management and analysis. We conducted a thematic analysis using the following steps: 1) data memoing: two analysts reviewed and memoed a subset of data to note emerging and repeated issues to facilitate code development; 2) code development: a codebook was developed comprising deductive codes from topics in the interview guides and inductive codes from memoing data; 3) coding data: we conducted inter-coder agreement to assess consistency between coders and discussed discrepancies before all transcripts were coded with codes from the codebook; 4) description of issues: a summary description of codes or groups of codes was made to begin describing and defining barriers and facilitators raised in the interviews; 5) comparisons: comparisons of barriers by participant characteristics were then conducted to identify any patterns by the participants’ demographics or other characteristics; 6) categorization of themes: description and comparisons of issues were used to categorize data into broader themes representing four core barriers (e.g., lack of GH exposure, lack of mentorship or support, realities of global work, and finances); and 7) conceptual framework: further analysis identified how each of the four barriers applied to the three stages of participants’ GH training (i.e., interest in GH, program application, and program participation). These results led to a framework (Figure 1) that shows how each barrier is present at various stages of participants’ training but operates differently. The conceptual framework was validated by reviewing data to ensure that all barriers and their alignment with different training stages are well supported by data.

### Quantitative data collection (phase 2).

We designed the quantitative survey based on the key objectives of the project and findings from the qualitative phase. Survey data were collected and managed using REDCap hosted at Emory University. All survey data were confidential and did not include personal identifiers. The link for completing the survey was shared with potential participants through various channels including e-mails to current and past consortia directors and program managers, especially for reaching FGHFS alumni and applicants, as well as targeted outreach through the National Medical Association, the American Public Health Association, and CUGH to identify potential qualified applicants. Electronic consent was obtained after ascertaining eligibility based on self-reported race–ethnicity criteria.

The survey instrument was in English and most of the questions were multiple choice with a few open-ended questions that were designed to elicit personal experience and recommendations. A single instrument was developed with appropriate branching logic and skip patterns that allowed different categories of respondents to complete the relevant sections. The survey instrument comprised 80 questions organized in the following sections: 1) demographics, 2) interest in GH, 3) FGHFS program application, and 4) FGHFS program participation (Supplemental Information 1). Response options for the survey questions were built on the four barriers identified in the qualitative phase so the survey could measure the extent and ranking of the barriers.

Questions for all respondents included when and where they were first exposed to GH, roles of key individuals who encouraged and/or discouraged them, and their specific areas of interest in GH. Additional questions for respondents who indicated they were interested in GH research included top challenges to pursuing a career in GH research. The section on the FGHFS program application included details regarding ease of application, and the section on FGHFS program participation included questions regarding program satisfaction, quality of mentoring, barriers/challenges faced during their training, and recommendations to promote GH research opportunities.

The survey was pretested by the study team, which included URM representation, and modified as appropriate to ensure completeness. The survey went live on November 15, 2023, and the data were locked on June 15, 2024, for analysis. All data were stored on a secure server and data cleaning and analyses were done using SAS.

## RESULTS

In the qualitative phase of the study, interviews were conducted with 18 participants including Group 1 (FGHFS Alumni, *n* = 7), Group 2 (FGHFS applicants, *n* = 3), and Group 3 (potential applicants, *n* = 8). Table 1 shows participant characteristics by group. To assess saturation (data adequacy), we reviewed data as they were collected; after 18 interviews, we found no new issues or nuances of issues being raised, so the sample size was adequate for our study goals.

**Table 1:** Participant characteristics—qualitative interviews

Characteristic	FGHFS Alumni (*n* = 7)	Applicants (*n* = 3)	Potential Applicants (*n* = 8)
	Number (%) or Mean (Range)	Number (%) or Mean (Range)	Number (%) or Mean (Range)
Gender			
Male	2 (29)	1 (33)	2 (25)
Female	5 (71)	2 (67)	6 (75)
Race			
Black	3 (43)	2 (67)	8 (100)
White	3 (43)	0	0
Pacific Islander	1 (14)	1 (33)	0
Ethnicity			
Hispanic/Latino	3 (43)	0	1 (12)
Non-Hispanic/Latino	4 (57)	3 (100)	7 (88)
Age*	33.5 (29–42)	34.3 (31–37)	32.6 (29–36)
Highest degree earned*			
MD	3 (43)	1 (33)	5 (72)
DVM	1 (14)	0	0
PhD	2 (29)	2 (67)	1 (14)
Master’s degree	0	0	1 (14)
Bachelor’s degree	1 (14)	0	0

DVM = Doctor of Veterinary Medicine; FGHFS = Fogarty Program for Global Health Fellows and Scholars; MD = Doctor of Medicine; PhD = Doctor of Philosophy.

* One participant in group 3 did not specify their age or highest degree earned.

In the quantitative phase of the study, 114 surveys were completed, of which 32 were ineligible as they did not meet the URM criteria based on self-reported race and ethnicity. Among the 82 eligible participants who completed the survey, there were 12 FGHFS alumni (Group 1), 10 FGHFS applicants (Group 2), and 60 potential applicants (Group 3). Key sociodemographic characteristics of the respondents are presented by group in Table 2. The majorities in all groups were female. Most self-identified as Black or African American, with higher proportions among applicants and potential applicants (80.0% and 81.7%) versus alumni (58.3%). Fewer potential applicants reported Hispanic ethnicity (16.7%) compared with the other groups (33% and 30%). Potential applicants were younger and more likely to be United States-born when compared with alumni and applicants. There were no major differences in parental education, but more alumni (83.3%) and applicants (70%) had completed graduate or postgraduate training compared with potential applicants (56.7%). Of the 80% of potential applicants who had heard about GH, 91% indicated that they were interested in GH, 63% were interested in GH research, and 23% were currently working in GH. The main areas of interest in GH were research, followed by community development and public health programs. Given that FGHFS is a research training program, all alumni and applicants were interested in GH research, and of these 75% and 70%, respectively, were currently working in GH. Alumni were more likely to report interest in research (100%) and less likely to report interest in GH patient care and health policy (42% and 33%) when compared with applicants and potential applicants. Most of the alumni and applicants who were currently working in GH also reported being situated in research or academia, whereas smaller proportions (10–20%) also reported volunteer/mission/nongovernmental organization engagement or government/public health sector work. Applicants were more likely to be working in healthcare.

**Table 2:** Participant characteristics—quantitative survey

	FGHFS Alumni (*n* = 12)	FGHFS Applicants (*n* = 10)	Potential Applicants (*n* = 60)
Gender			
Male	3 (25.0%)	2 (20.0%)	5 (8.3%)
Female	7 (58.3%)	6 (60.0%)	48 (80.0%)
Declined to answer	2 (16.7%)	2 (20.0%)	7 (11.7%)
Age (years)			
Mean (SD)	39.0 (7.0)	36.1 (5.3)	31.4 (8.0)
18–30	1 (8.3%)	1 (10.0%)	31 (51.7%)
31–45	8 (66.7%)	9 (90.0%)	25 (41.7%)
>46	3 (25.0%)	0 (0.0%)	3 (5.0%)
Missing	0 (0.0%)	0 (0.0%)	1 (1.7%)
Race			
White	2 (16.7%)	1 (10.0%)	5 (8.3%)
Black or African American	7 (58.3%)	8 (80.0%)	49 (81.7%)
2 races: Black or African American and Asian/white	0 (0.0%)	1 (10.0%)	3 (5.1%)
2 races: American Indian and white	1 (8.3%)	0 (0.0%)	0 (0.0%)
3 races: white, Black or African American, and American Indian/Caribbean	1 (8.3%)	0 (0.0%)	1 (1.7%)
Unknown	1 (8.3%)	0 (0.0%)	2 (3.4%)
Ethnicity			
Hispanic	4 (33.3%)	3 (30.0%)	10 (16.7%)
Non-Hispanic	8 (66.7%)	6 (60.0%)	50 (83.3%)
Decline to answer	0 (0.0%)	1 (10.0%)	0 (0.0%)
Education			
College (4-year degree)	0 (0.0%)	1 (10.0%)	19 (31.7%)
Graduate	1 (8.3%)	3 (30.0%)	21 (35.0%)
Postgraduate (residency or fellowship)	9 (75.0%)	4 (40.0%)	13 (21.7%)
Missing data	2 (16.7%)	2 (20.0%)	7 (11.7%)
Country of birth			
United States	5 (50.0%)	1 (10.0%)	41 (68.3%)
Canada	0 (0.0%)	1 (10.0%)	0 (0.0%)
Mexico	0 (0.0%)	0 (0.0%)	1 (1.7%)
Nigeria	2 (20.0%)	2 (20.0%)	2 (3.3%)
Others	3 (30.0%)	4 (40.0%)	9 (15.0%)
Missing	2 (16.7%)	2 (20.0%)	7 (11.7%)
Mother’s country of birth			
United States	3 (30.0%)	0 (0.0%)	24 (40.0%)
Mexico	0 (0.0%)	0 (0.0%)	2 (3.3%)
Nigeria	3 (30.0%)	3 (30.0%)	12 (20.0%)
Others	4 (40.0%)	5 (50.0%)	15 (25.0)
Missing	2 (16.7%)	2 (20.0%)	7 (11.7%)
Father’s country of birth			
United States	1 (8.3%)	0 (0.0%)	24 (40.0%)
Mexico	0 (0.0%)	0 (0.0%)	2 (3.3%)
Nigeria	3 (25.0%)	3 (30.0%)	12 (20.0%)
Others	6 (50.0%)	5 (50.0%)	14 (23.3%)
Do not know	0 (0.0%)	0 (0.0%)	1 (1.7%)
Missing	2 (16.7%)	2 (20.0%)	7 (11.7%)
Mother’s education			
High school or less	4 (40.0%)	2 (20.0%)	7 (11.7%)
College (2-year degree)	1 (10.0%)	0 (0.0%)	8 (13.3%)
College (4-year degree)	0 (0.0%)	4 (40.0%)	16 (26.7%)
Graduate	5 (50.5%)	2 (20.0%)	22 (36.7%)
Missing	2 (16.7%)	2 (20.0%)	7 (11.7%)
Father’s education			
High school or less	4 (33.3%)	1 (10.0%)	15 (25.0%)
College (2-year degree)	2 (16.7%)	0 (0.0%)	3 (5.0%)
College (4-year degree)	0 (0.0%)	4 (40.0%)	14 (23.3%)
Graduate	2 (16.7%)	2 (20.0%)	18 (30.0%)
Postgraduate (residency or fellowship)	2 (16.7%)	1 (10.0%)	2 (3.3%)
Unknown	0 (0.0%)	0 (0.0%)	1 (1.7%)
Missing	2 (16.7%)	2 (20.0%)	7 (11.7%)

*n* = % unless specified otherwise.

### Training stage 1: Interest in global health.

Qualitative in-depth interviews revealed that all participant groups felt their interest in a GH career was curtailed by their lack of exposure to GH, global research or international fieldwork (see Figure 1/barrier 1). The quantitative survey revealed that 20% of potential applicants had not heard of GH. Nearly half (47%) of the respondents who had heard about GH indicated that that they had first heard about it when they were 15–20 years of age, followed by a quarter (26%) who reported exposure during ages 21–25. Most respondents (75%) reported that their first exposure to GH occurred during college/university, medical school, or postgraduate studies. The next most reported exposure was from family followed by community organizations. Few survey respondents (<20%) selected “media” or “high school.” When asked about sources of influence to pursue a career in GH research, more than half of respondents reported college/university, medical school, or postgraduate studies, followed by “personal interest/feeling/aspiration” and/or “family background.” Nearly a third also reported personal international travel experiences.

**Figure 2.**
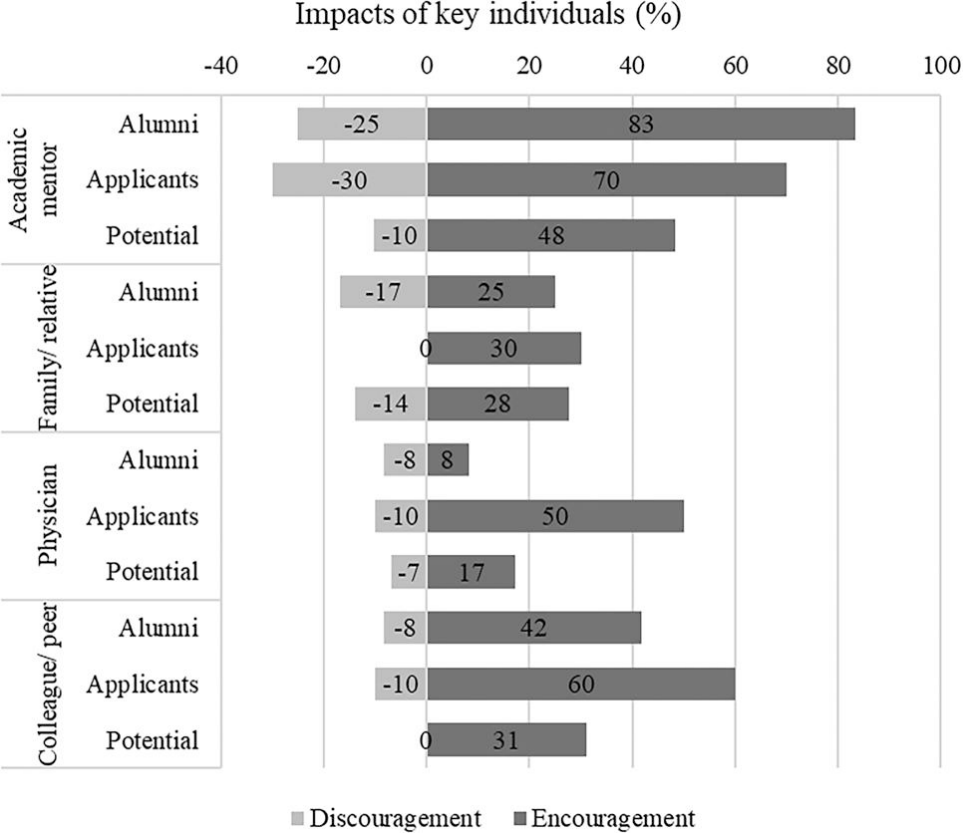
Key individuals influencing respondents’ interest in global health (GH), by participants’ training stage, from our qualitative and quantitative studies.

The quantitative survey permitted an assessment of key individuals who either encouraged or discouraged the pursuit of a career in GH research. These are shown by group in Figure 2. For all groups, academic mentors, followed by colleagues or peers and physicians, provided the most encouragement. Alumni were more likely to report that academic mentors encouraged them, compared with applicants or potential applicants. However, the percentage of those who received negative guidance from these same groups was not insignificant, particularly in the case of mentors (Figure 2).

This critical importance of mentors impacted careers in GH at all stages. In the in-depth interviews, participants reported that a lack of mentoring in GH dampened their interest (Figure 1/barrier 2). They described a distinct absence of role models and mentors in GH from historically underrepresented groups who could provide inspiration and guidance to navigate a GH career. For example:

“*…they brought in professionals that are doing this type of GH work, and I felt like there were not enough historically underrepresented researchers that were part of giving those. I want to see people that look like me or that are part of historically underrepresented groups giving these presentations, not just people that are typically overrepresented and in this sort of way.”—Group 1 in-depth interview (IDI) 6*

Participants stated that they often transitioned into GH from other disciplines (e.g., epidemiology, medicine, or medical specialties like cardiology, internal medicine, orthopedics, or veterinary medicine), but they received little mentoring to effectively make this shift and overcome many challenges specific to GH. This was a deterrent to moving into a GH career.

**Figure 3.**
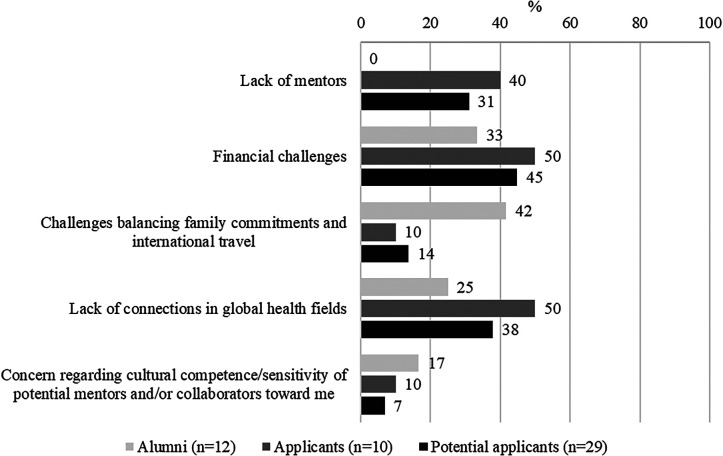
Principal challenges to the pursuit of global health training opportunities encountered by study participants, by training stage (*n* = 51).

Participants’ interest in GH work was compromised by various other perceived challenges (Figure 1/barrier 3 and Figure 3). Some described travel-related burdens as a challenge, including travel logistics (e.g., visas, flights, time zones), field work in overseas locations, and safety abroad for women. Participants also felt the regular separation from family while working abroad was a challenge, because it required a supportive family/spouse to manage their home life while they are working abroad (Figure 1/barrier 3). This was a particular concern among women of all backgrounds and those with children, who referenced societal/cultural expectations for women to curtail their careers to raise their families. This created a moral struggle among participants to choose between family obligations and travel abroad for a GH career. Many of these concerns were identified as actual issues by those working in GH, thus permeating all three stages of GH training that we studied.

### Training stage 2: FGHFS program applicants.

The critical importance of mentors and lack of encouraging influences persisted as a barrier for participants when applying to the Fogarty GH program (Figure 1/barrier 2). Participants described work colleagues, professors, and mentors outside of GH being unsupportive of their application to GH programs, stating that, without a Master of Public Health or global experience, they may not succeed. Friends and family members also discouraged some from applying because of concern about their safety while working abroad, particularly for women (Figure 1/barrier 3). Some participants were discouraged by a lack of support of their family, and felt they may disappoint them by not pursuing a more traditional career option such as an office job with a stable work schedule, whereas GH requires greater flexibility and frequent international travel. Family members also expressed concern about the lower pay of GH work and preferred them to choose more culturally expected careers with higher salaries and greater prestige (e.g., medical doctor or specialist) (Figure 1/stage 1/barrier 3). The influence of such opinions in either encouraging or discouraging an individual’s interest in a GH career was redemonstrated in the quantitative survey results (Figure 2).

Fellowship applicants also reported a lack of transparency on the amounts of their fellowship stipends (Figure 1/barrier 4). This heightened their prior concerns about the inadequate salaries of GH positions. In addition, they felt there was limited guidance on financing their fellowship, which was exacerbated when trying to develop a budget for their application. These concerns were worsened by a lack of program personnel to provide guidance on financial aspects of participating in the Fogarty GH program.

Qualitative interviews also revealed that most participants had no experience with writing research grant applications, seeking ethical approval, data collection, or working in international settings, which they felt would limit their success in a GH career (Figure 1/barrier 1). Academic settings and procedures, professional practices, and related social norms were unfamiliar and daunting to many participants. This created self-doubt and feelings of inadequacy about their ability to succeed in the academic environment of GH. They also had limited connections in GH, which they perceived as an impediment to success. These limited connections were cited as an important barrier to GH careers across all participants at all training stages but seemed more relevant among applicants. With limited research experience, many felt confused and lacked confidence to develop an effective fellowship application (e.g., writing an academic research proposal, finding mentorship, making a budget, etc.) and found the application process to be overly complicated. This led to feelings of doubt in the strength of their application and the likelihood of acceptance, which became a deterrent to future applications.

Three fellowship applicants who participated in the quantitative survey reported that the application was too long and intensive and/or they had difficulties writing a research proposal or collecting reference letters or had financial concerns.

The importance of mentors in overcoming career path difficulties and writing an effective application was reinforced by the survey, which found that more than 90% of the alumni reported contacting their mentor for guidance with the application process. They considered doing so the most important reason for their success in being accepted in the training program, followed by 42% who also reported consulting with a Fogarty or funding source representative and/or starting early; 33% also got help from others besides their mentor. When asked about ease of the FGHFS application process, 42% of the alumni reported that it was moderately easy, whereas the rest stated that it was moderately difficult (33%) or had neutral opinions (25%).

The most common reason in the quantitative survey for not applying to the Fogarty GH training program by potential applicants who were interested in GH research (*n* = 29) was lack of awareness of the opportunities (63%), followed by being told or assuming they were not qualified (11%) and/or they could not commit the required time to participate in the program (7%).

### Training stage 3: program participation (FGHFS alumni).

For those who did apply and participate in FGHFS, feelings of inadequacy were perpetuated by their lived experiences in the program (Figure 1/stage 3/barrier 3). Participants described experiences where a supervisor or colleague did not acknowledge or respect their cultural identity or the culturally significant aspects of their work. Concern over lack of cultural competence of colleagues in the field was a further deterrent to GH work. These sentiments were echoed in our quantitative survey, which revealed that 25% of participants reported lack of connection in GH fields and 17% reported concern regarding the cultural competence/sensitivity of potential mentors and/or collaborators toward them (Figure 3).

Participants described a bias against their ability to succeed in GH, and discrimination when working abroad, whereby they felt they were treated unfairly based on their race and differing cultural background. This challenge disproportionately affected African American participants who experienced discrimination, whereas those working in their home country or internationally but among similar race/ethnic communities did not encounter discrimination. Furthermore, participants who worked across cultures described some cultural barriers to doing GH work, such as adjusting to cultural norms while working abroad and experiencing culture shock (Figure 1/barrier 3). Some described uncertainty on how to dress or interact respectfully with others while working in cultures very different from their own. These challenges heightened feelings of isolation and uncertainty in GH work. For example:

“*I think just about American culture, that kind of link back to my own personal identities, and it would have been nice to have some primer of what that looks like there, and what people expected and how I should interact or should not with certain people. What attire is appropriate to wear in a business setting or not, because in India there’s, you know, a lot of regional variation, and in clinical settings versus community, that’s different. And what’s formal and not? There’s like a thousand iterations. So, I think a lot of the challenges on the day to day were around my identity and tied back to these kinds of social nuances.”—Group 1 IDI 3*

Participants also described wrestling with working in a modern GH sphere, whereas others raised concerns about being part of a colonialist system that associates GH with Western dominance. For example:

“*I mean, there’s this thing happening in the sector right now, decolonization. And what that means, and where that leaves people like me, I’m definitely on board with that agenda, but it also brings into question what the role for someone like me is in GH. So, I think that it’s not one person who has been discouraging me to be a part of GH, but it’s also me asking myself, what is my role?”—Group 2 IDI 2*

Such issues led participants to describe experiencing “imposter syndrome,” i.e., their limited experience led to feelings that they did not belong in GH or in an academic setting (Figure 1/barrier 1). Many participants had self-doubt and questioned whether they were qualified enough for academic work, or whether their skills were sufficient when compared with non-URM colleagues whom they viewed as more experienced. For example:

“*The biggest challenge? I feel like imposter syndrome is definitely one of the larger ones… especially being the only underrepresented minority, knowing that I don’t have a lot of insight in regard to what I’m doing. Aside from the things that my mentor shared with me, which I feel like I didn’t really pick up until I was in the fellowship. So before and during the fellowship, I didn’t really know as much as I felt like everyone else did, even when we were meeting, even when I was submitting my applications and stuff, I felt like I was just always questioning what I was doing, if it was enough.” —Group 1 IDI 2*

Feelings of not belonging were compounded by the lack of URM peers during their participation in FGHFS, which led to feeling isolated in several ways. They described a lack of URM peers in the program with whom they could relate, find camaraderie, and receive emotional support. This led to feeling lonely and disconnected from their peer group while abroad. Although they were aware of their peers’ career goals, they were reluctant to ask about them because of feeling like outsiders (Figure 1/barrier 2).

**Figure 4.**
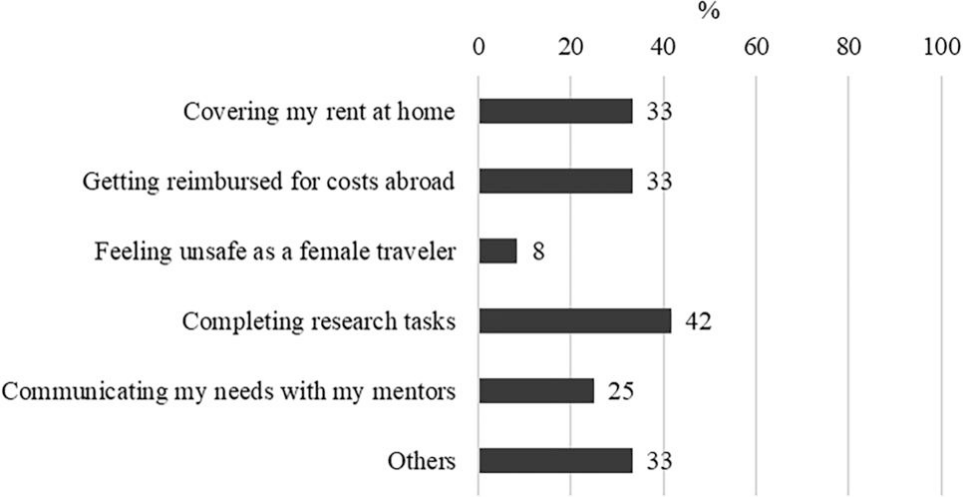
Principal challenges faced by alumni who had participated in the Fogarty Program for Global Health Fellows and Scholars (FGHFS) (*n* = 12).

Feelings of isolation were also caused by a lack of guidance and institutional support from in-country mentors, to navigate working in a GH setting as a URM individual. This concurred with findings in the quantitative assessment in which less than half of the respondents rated mentoring at the LMIC site as very good/moderately good (41.7%/42%), in contrast to mentoring from the parent site for which the quality of mentoring was very good/moderately good (58%/17%). Additional challenges reported by a minority of alumni included communicating needs with mentors, completing research tasks, and financial issues such as getting reimbursed for costs abroad and covering rent at home (Figure 4).

All groups in the survey reported finances as a challenge to pursuing GH research (Figure 3). Financial concerns were also raised as a barrier to GH careers in the qualitative interviews, including lack of familiarity with funding mechanisms for GH research (Figure 1/stages 1 and 3/barrier 4). Participants described not knowing how to acquire research funding or the amount of funding needed to conduct international research or how to budget salary for international research staff. Another concern was insufficient pay for GH positions. Many URM participants were financially supporting their immediate and extended family members and were concerned that the salary of a GH position would be insufficient to support their financial needs. Additionally, URM participants expressed concerns that they would not be sufficiently compensated for their work based on prior experiences where they were underpaid or uncompensated (e.g., to gain experience, not paid as a postdoctoral researcher, working overtime without additional pay).

Participants expressed concerns about covering personal expenses both abroad and at home while conducting global fieldwork, e.g., paying rent both at home and abroad, paying bills on time, and financially supporting family while abroad (Figure 4). Additionally, some participants described their research interests as community-based and focused on developing interventions, which they felt were often not attractive to mainstream funding organizations like the NIH. In contrast, they felt that their peers who conduct bench science research were more easily funded (Figure 1/barrier 4). They described not being aware of this funding challenge until they were immersed in GH fieldwork. For example:

“*In general, folks in my background aren’t really classically drawn to fundable research… It’s all sort of wanting to make an impact in the community in a real way and a tangible way. I know that bench research makes a real difference, but I just think folks from my background, in general, are more interested in how do I improve health outcomes for our population? How do I improve health outcomes in the area that I’m from? How do I keep you from dying young or unnecessarily, right? How do I improve what now is termed health equity. Right? And those aren’t things that are classically funded by NIH.”—Group 1 IDI 7*

While enrolled in a global heath program, participants described challenges balancing their family needs with career obligations (Figure 3). Identified as an initial concern during the interest stage, it became more complicated when conducting GH work because of unanticipated challenges that arose (Figure 1/stages 1 and 3/barrier 3). This amplified participants’ stress, particularly among women who continued to feel pressure to sacrifice their careers to care for family.

**Figure 5.**
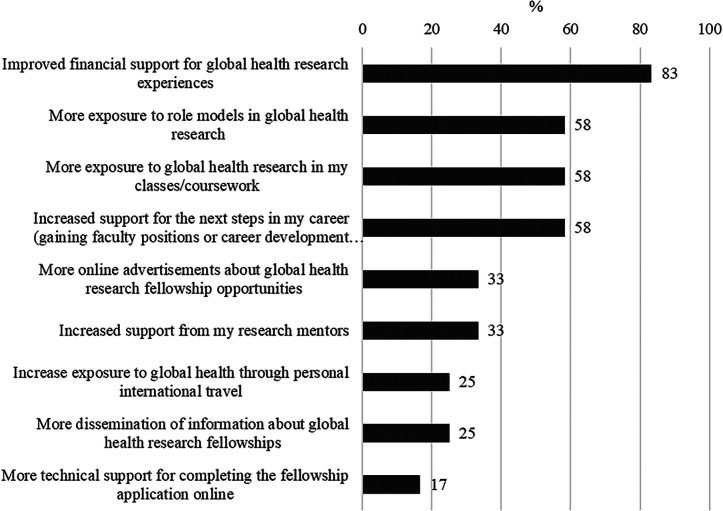
Fogarty Program for Global Health Fellows and Scholars (FGHFS) alumni recommendations for recruiting URM global health research trainees (*n* = 12).

Alumni also provided several recommendations for recruiting more URM GH research trainees in the survey (Figure 5). The most common recommendations were to improve financial support for GH research experiences, followed by increased exposure to GH research in classes/course work and to role models in GH research, and increased support for next steps in their careers. Open-ended survey responses also included recommendations such as providing financial support to underrepresented/minority groups, to early career researchers to initiate new projects, and/or for those with family commitments or other constraints (both personal and career-based); improving mentorship support by increasing minority representation among mentors/leaders to improve cultural sensitivity and training experience and easily accessible mentors; and finally, to have targeted GH outreach to underrepresented groups and to call for university and educational systems to increase GH education and career opportunities in general.

## DISCUSSION

Our exploratory sequential mixed-methods study of historically underrepresented groups in a GH research training program identified several barriers, as well as potential facilitators to increase participation that led to the development of a conceptual framework that is supported by both our qualitative and quantitative data (Figure 1). Each of four principal barriers operates independently in the three stages of training, and the simultaneous presence of multiple barriers for an individual at any given stage amplifies the challenges faced by a URM seeking GH training. The effects of a given barrier also became more pronounced as an individual’s GH training progressed. The cumulative sequential effect of a barrier or combination of barriers over the course of a career may have profound deterring effects on one’s pursuit of GH.

We found that academic mentors played strong roles, mainly providing encouragement but at times discouraging URMs from pursuing GH careers. Other key individuals, namely family members, physicians, and colleagues or peers, also influenced participants’ decisions. Major challenges included lack of mentors, insufficient connections to GH fields, and financial barriers. Bias, lack of cultural competence, and cultural insensitivity of mentors or collaborators were also issues. Lack of family support; discouragement from peers, friends, and mentors; and inexperience with international travel were also barriers. Persons who had successfully pursued GH training opportunities were largely satisfied with their experiences but were challenged by financial needs such as covering their rent at home, by timely reimbursement for costs while abroad, and, for female alumni, by concerns about international safety.

The principal recommendations from successful program alumni for recruiting more URM GH research trainees included providing greater exposure to GH role models, more relevant course material in their academic studies, and focused career advising, as well as more robust financial support. Other suggestions to address the barriers faced by URMs included providing guidance to mentors, earlier exposure to GH, community work, and increasing access to academic mentors, mental health resources, and financial advisors.

Our findings show that diversity in the GH workforce is challenged by a range of systemic barriers. The underrepresentation of URMs in higher education, particularly science-related fields, affects the eligibility of URMs to enter GH, where a graduate degree is often required. For example, only 6% of the United States medical workforce are Black/African American, despite representing 13% of the United States population.^13^ The limited representation of women and minorities in science, technology, engineering, and mathematics (STEM) disciplines is because of limited resources for further education and financial constraints because of family responsibilities.^5^ Lack of family support has also been reported as a deterrent to pursuing a GH scientific career. URM students, particularly those who are first-generation college students, have reported concerns from family members about pursuing higher education versus seeking employment.^14^ Similar barriers to participating in GH research were reported by all the groups in both our qualitative interviews and survey. These concerns, especially financial barriers, also deter people from URM backgrounds from entering GH fields because financial aid is limited for GH training programs, making them less accessible for those with limited financial resources. We found that personal and professional connections were important, similar to earlier reports that showed that URM students lacked personal and professional connections to pursue graduate-level STEM studies because of disconnects between their communities and the academic environment.^14^ This is also supported by our finding that lack of mentors was reported as a key constraint by potential applicants and by persons who applied but did not participate in FGHFS, compared with FGHFS alumni who benefited from academic mentors who encouraged them. Finally, GH leadership positions are predominantly held by white Americans, which may limit opportunities for URMs.^4^ Like Mays et al.,^5^ we found that lack of knowledge about GH careers and training opportunities was an important barrier that needs to be addressed by more inclusive policies and expanding diversity at higher levels of GH leadership in diverse settings including academia.

Communities tend to trust services from people who they believe have similar personal beliefs, values, and ways of communicating.^15^ A more diverse GH workforce can help to address healthcare disparities that impact minority groups, by creating a GH workforce that enhances cross-cultural competencies and provides more inclusive healthcare at the international level.^16^^,^^17^ Therefore, many racial and ethnic minority organizations emphasize a need for more minority representation in healthcare and GH work.^17^ This includes having more GH professionals work within their home countries or with those of a similar race or background to themselves.^18^

Interventions to promote involvement of people from URM backgrounds in healthcare fields have included encouraging early exposure to healthcare careers through volunteering, clinical, and clerical experiences, starting with involvement in college student organizations such as minority healthcare groups or collaboration with faculty to increase exposure to healthcare among those from URM backgrounds.^19^^,^^20^ Other interventions include promoting involvement in cultural and medical mission trips to introduce GH careers to people from URM backgrounds, allowing them to connect to their culture while exploring scientific career paths.^21^^,^^22^ Scholarships, mentoring, and URM-specific internships are further initiatives to encourage participation of people from URM backgrounds in health professions and further training.^22^^,^^23^ However, despite these efforts to increase diversity, health professionals remain less diverse than the populations they serve.^4^ Although previous studies have identified barriers for URMs in pursuing higher education and careers in STEM fields, they offer only broad reasons for the lack of diversity in GH. Research on the lack of diversity in the GH workforce and its causes is limited, partly because of the small number of GH training programs in the United States, and no study to our knowledge has examined barriers in depth from the perspective of URMs themselves. Furthermore, previous research provides no insight on when specific barriers occur and how they operate in detail.^4^

### Strengths and limitations.

The use of an exploratory sequential mixed-methods study design is a major strength of our study. We also included URMs at different stages, namely individuals who expressed interest in GH but had not applied for GH training programs, those who had applied for a program but were not admitted, and alumni of FGHFS. A robust analysis of our qualitative findings was supported by the results of a quantitative survey.

Some important limitations remain. The definition of URM can be challenging. We relied on self-identification but obtained information on the countries of birth of the respondents’ parents, to address differences between those who are first- or second-generation migrants compared with multigenerational African Americans with their unique history and experiences. Sample size was a limitation especially for the survey, which affects validity, generalizability, and ability to carry out formal statistical testing of the survey data. For the qualitative phase, we also had a small number of FGHFS applicants who did not end up participating in FGHFS. Comparison to experiences of non-URM groups was beyond the scope of the study but would have provided another level of analysis. Likewise, we lack data from other key stakeholders such as application reviewers, NIH program officials, or other relevant administrators.

## CONCLUSION

Our findings suggest that key barriers faced by URMs to pursuing GH careers include lack of exposure to GH careers, issues related to GH work, lack of mentors, and financial challenges. These barriers may be compounded when URM individuals move from interest in GH to applying for a learning opportunity to participation in a GH program. Our qualitative findings also identified lack of experience in GH as a barrier. URMs who successfully participated in FGHFS may have been exposed to GH earlier and typically had GH-focused mentors who exposed them to GH opportunities and assisted them in the application process. Future efforts should address these barriers systematically to increase URM engagement in GH training opportunities.

## Supplemental Materials

10.4269/ajtmh.24-0847 Supplemental Materials

## References

[1] Griffin K Baker V O’Meara K Nyunt G Robinson T Staples CL, 2018. Supporting scientists from underrepresented minority backgrounds: Mapping developmental networks. Stud Grad Postdr Educ 9: 191–37.

[2] NSF, 2019. Women, Minorities, and Persons with Disabilities in Science and Engineering: 2019. Alexandria, VA: National Science Foundation.

[3] NIH, 2024. Underrepresented Groups. Available at: https://extramural-diversity.nih.gov/diversity-matters/underrepresented-groups. Accessed December 15, 2024.

[4] Coronado F Beck A Shah G Owens-Young J Sellers K Leider J, 2019. Understanding the dynamics of diversity in the public health workforce. J Public Health Manag Pract 26: 389–392. 10.1097/PHH.0000000000001075 PMC7190406 31688743

[5] Mays D Klaiman T Kumanyika S Bernhardt JM, 2008. A call to action to address diversity in public health professional preparation. DEHC 5: 207–214.

[6] Beaglehole R Bonita R, 2010. What is global health? *Glob Health Action* 3. doi: 10.3402/gha.v3i0.5142. PMC2852240 20386617

[7] Ba M Gebremedhin LT Masako P Msigallah F Kone KE Baird TL, 2021. Diversity and solidarity in global health. *Lancet Glob Health* 9: e391–e392. 33609482 10.1016/S2214-109X(21)00029-2

[8] USDHHS, 2001. National Standard for Culturally and Linguistically Appropriate Services in Health Care. Washington DC: PHSD, Office of Minority Health.

[9] FIC, 2024. Global Health Program for Fellows and Scholars. Available at: https://www.fic.nih.gov/Programs/Pages/scholars-fellows-global-health.aspx. Accessed April 8, 2025.

[10] Harris PA ; REDCap Consortium, 2019. The REDCap consortium: Building an international community of software platform partners. J Biomed Inform 95: 103208. 31078660 10.1016/j.jbi.2019.103208 PMC7254481

[11] Harris PA Taylor R Thielke R Payne J Gonzalez N Conde JG, 2009. Research electronic data capture (REDCap)–a metadata-driven methodology and workflow process for providing translational research informatics support. J Biomed Inform 42: 377–381. 18929686 10.1016/j.jbi.2008.08.010 PMC2700030

[12] VERBI Software, MAXQDA 2022. Berlin, Germany: VERBI. Available at: https://www.maxqda.com. Accessed April 11, 2025.

[13] AAMC, 2019. Diversity in Medicine: Facts and Figures 2019. Washington, DC: Association of American Medical Colleges, AAMC.

[14] Holley KA Gardner S, 2012. Navigating the pipeline: How socio-cultural influences impact first-generation doctoral students. J Divers High Educ 5: 112–121.

[15] Street RL KJ O’Malley Cooper LA Haidet P, 2008. Understanding concordance in patient-physician relationships: personal and ethnic dimensions of shared identity. Ann Fam Med 6: 198–205. 18474881 10.1370/afm.821 PMC2384992

[16] Garces LM Mickey-Pabello D, 2015. Racial diversity in the medical profession: The impact of affirmative action bans on underrepresented student of color matriculation in medical schools. J High Educ 86: 264–294. 10.1353/jhe.2015.0009 PMC4454423 26052161

[17] Glass R, 2019. Diversity Strengthens Global Health Science. Available at: https://www.fic.nih.gov/News/GlobalHealthMatters/july-august-2019/Pages/roger-glass-opinion-diversity-strengthens-global-health-science.aspx. Accessed December 18, 2024.

[18] NIHB, 2020. National Indian Health Board and Public Health. Available at: https://www.nihb.org/public_health/public_health.php. Accessed December 15, 2024.

[19] Muncan B Majumder N Tudose N, 2016. From high school to hospital: How early exposure to healthcare affects adolescent career ideas. *Int J Med Educ* 7: 370–371. 27816962 10.5116/ijme.5801.f2cc PMC5116365

[20] Rumala BB Cason FD Jr. 2007. Recruitment of underrepresented minority students to medical school: Minority medical student organizations, an untapped resource. J Natl Med Assoc 99: 1000–1004. 1008–1009. 17913109 PMC2575864

[21] Martin TE Parker LM Mugambi CM, 2019. The impact of an international medical mission trip on the cultural competency of healthcare providers. J Cult Divers 26: 76–51.

[22] Vu MT Johnson TR Francois R Simms-Cendan J, 2014. Sustained impact of short-term international medical mission trips: Resident perspectives. *Med Teach* 36: 1057–1063. 25072942 10.3109/0142159X.2014.920491

[23] Cole DC , 2016. Mentoring health researchers globally: Diverse experiences, programs, challenges, and responses. Glob Public Health 11: 1093–1108. 26234691 10.1080/17441692.2015.1057091 PMC5020346

