# Supplementary material for: Barriers and Facilitators for Participation in Global Health Research Training Programs Among Underrepresented Minority Groups
Source: Am J Trop Med Hyg. 2025 May 13;113(1):223–33. doi: 10.4269/ajtmh.24-0847 (PMC12225555; doi:10.4269/ajtmh.24-0847)
Supplement: Supplemental Materials [file tpmd240847.SD1.pdf]

EDGE SURVEY - November 2023

Please complete the survey below.

Thank you!

A major goal of the NIH Fogarty International Center (FIC) is to foster the next generation of global health scientists from the US and low- and middle-income countries (LMICs). US-based university consortia, in partnership with their LMIC scientific collaborators, provide mentored LMIC-based global health research training opportunities for science and health professions trainees. Despite concerted efforts by FIC and shared dedication of the consortia to promoting diversity and inclusion, few persons from US historically underrepresented groups (HUGs) have participated in the offered training opportunities. We aim therefore to systematically identify and evaluate barriers and develop innovative strategies to improve HUGs participation in global health research training opportunities. Your completion of this survey will help us understand how we may accomplish these goals.

This survey is approved by the Institutional Review Board for Human Use of Emory University. All responses will be entirely anonymous, and the data will be stored securely and confidentially. No compensation will be provided for completion of the survey. We anticipate that the survey should take between 10 and 30 minutes to complete. Completion of the survey constitutes your indication that you have provided informed consent.

A. DEMOGRAPHICS

Please tell us a few things about yourself.

What is your age?

____

Which ethnicity do you identify with? (choose one)

- ◯ Hispanic or Latino
- ◯ Non-Hispanic or Latino
- ◯ Unknown
- ◯ Decline to answer

Which race(s) do you identify with? (check all that apply)

- ☐ White
- ☐ Black or African American
- ☐ American Indian
- ☐ Asian
- ☐ Native Hawaiian / Pacific Islander
- ☐ Prefer not to answer
- ☐ Other (please specify)

Please specify other

____

B. INTEREST IN GLOBAL HEALTH

The following questions ask about your knowledge of and exposure to global health.

Have you heard about global health?	◯ Yes<br>◯ No
Where were you first exposed to the field of global health? (check all that apply)	☐ My family/relatives<br>☐ My family background<br>☐ During High school<br>☐ During college/university/medical school/post grad<br>☐ In my religious institution(s)<br>☐ Through my community organizations<br>☐ In the media (this includes news, entertainment, TV, internet)<br>☐ Other (specify)
Please specify other	__________________________________
Would you describe yourself as interested in global health?	◯ Yes<br>◯ No
Which of the following areas are you interested in? (check all that apply)	☐ Health Policy<br>☐ Research<br>☐ Patient Care<br>☐ Community Development<br>☐ Public health Programs<br>☐ Other (specify)
Please specify other	___________________________
At what age (years) did you first develop an interest in global health?	◯ Under 10<br>◯ 10-14<br>◯ 15-20<br>◯ 21-25<br>◯ 26-30<br>◯ 31-35<br>◯ Over 35<br>◯ Not sure
Are you currently working in global health?	◯ Yes<br>◯ No
If yes, what type of position do you have? Check all that apply	☐ Academic<br>☐ Research<br>☐ Government<br>☐ NGO<br>☐ Private sector<br>☐ Health care<br>☐ Volunteer/mission<br>☐ International agency<br>☐ Public health sector<br>☐ Other (specify) [free text]
Please specify other	___________________________

Have you ever considered pursuing a career in global health research?	◯ Yes<br>◯ No
Why not?	__________________________________
What influenced you to consider a career in global health research? (check all that apply)	☐ Journals/magazine<br>☐ Personal international travel<br>☐ Media (social, television, celebrities)<br>☐ Personal interest/feeling/aspiration<br>☐ Family background<br>☐ College/university/medical school/post grad<br>☐ Other (specify)
Please specify other	___________________________
Did any of the following persons encourage you to consider a career in global health research? (check all that apply)	☐ Academic mentor (teacher/advisor)<br>☐ Family / relative<br>☐ Physician<br>☐ Neighbor/friend<br>☐ Colleague/peer<br>☐ Other (specify)
Please specify other	___________________________
Did any of the following persons discourage your decision to consider a career in global health research? (check all that apply)	☐ Academic mentor (teacher/advisor)<br>☐ Family / relative<br>☐ Physician<br>☐ Neighbor/friend<br>☐ Colleague/peer<br>☐ None of the above
Did you experience any challenges in pursuing your interest in a career in global health research?	◯ Yes<br>◯ No
What challenges did you experience in pursuing your interest in a career in global health research? (check all that apply)	☐ Lack of family support<br>☐ Lack of research experience<br>☐ Lack of mentors<br>☐ Lack of HUGs specific mentors<br>☐ Discouragement from others (peers, friends, mentors)<br>☐ Financial challenges<br>☐ Challenges balancing family commitments and international travel<br>☐ Minimal prior experience with international travel<br>☐ Lack of connections in global health fields<br>☐ Concern regarding cultural competence/sensitivity of potential mentors and/or collaborators toward me<br>☐ Other (specify and/or elaborate on options checked)
Please specify other	__________________________________

Identify the most significant challenges you faced in pursuing a career in global health research (up to three)	☐ Lack of family support<br>☐ Lack of mentors<br>☐ Discouragement from others (peers, friends, mentors)<br>☐ Financial challenges<br>☐ Challenges balancing family commitments and international travel<br>☐ Minimal prior experience with international travel<br>☐ Lack of connections in global health fields<br>☐ Concern regarding cultural competence/sensitivity of potential mentors and/or collaborators toward me<br>☐ Other (Specify and/or elaborate on options checked)
Please specify other	__________________________________
Have you ever applied to any global health research training program?	◯ Yes<br>◯ No
Why have you not applied to any global health research training program?	◯ I was not aware of opportunities<br>◯ I was told I was not qualified or assumed I was not qualified<br>◯ Couldn't commit the required time to participating in the program<br>◯ The application process was difficult or complicated<br>◯ Other (Specify)
Please specify other	____________________________
To which global health research programs(s) did you apply? (check all that apply)	☐ Fulbright<br>☐ Fulbright/Fogarty Training Programs<br>☐ Fogarty Global Health Fellows and Scholars (including FICRS-F or LAUNCH)<br>☐ Other (specify)
Please specify other	____________________________

C. PROGRAM APPLICATION

The following questions ask about your experience applying for a global health research training program.

How easy or difficult did you find the application process?

◯ Very easy
◯ Moderately easy
◯ Neutral
◯ Moderately difficult
◯ Very difficult

Did you face any challenges in completing the program application?

◯ Yes
◯ No

What challenges did you face? (check all that apply)

☐ Downloading the application
☐ Application was not user friendly
☐ Application took a lot of time to complete
☐ Collecting letters of support
☐ Lack of familiarity with developing a research proposal
☐ Financial concerns
☐ Difficulty acquiring the needed information
☐ Lack of support or lack of guidance on completing the application
☐ Other (specify and/or elaborate on options checked)

Please specify other

____

What would have made the application process easier for you?

____

Did you submit an application?

◯ Yes
◯ No

Why did you not submit an application?

____

What do you think was key to successfully submitting your application? (check all that apply)

☐ I started early.
☐ I consulted with a Fogarty/other funding source representative
☐ I contacted my mentor for guidance
☐ I received help from others (other than my mentor)
☐ I took time off to do the application
☐ Other (specify)

Were you awarded the fellowship?

◯ Yes
◯ No

Why do you think you were not awarded the fellowship?

____

Did you accept the fellowship?

◯ Yes
◯ No

Why did you not accept the fellowship?

____

D. PROGRAM PARTICIPATION

The following questions relate to your experience in the global health research training program in which you enrolled.

How would you rate your overall experience in the program?

- ◯ Very satisfied
- ◯ Moderately satisfied
- ◯ Neutral
- ◯ Moderately dissatisfied
- ◯ Very dissatisfied

Did you experience any of the following during your training? (Check all that apply)

- ☐ Difficulty covering my rent at home
- ☐ Challenges getting reimbursed for costs abroad
- ☐ Feeling unsafe as a female traveler
- ☐ Difficulty completing research tasks
- ☐ Difficulty communicating my needs with my mentors
- ☐ Other (specify and/or elaborate on options checked)

Please specify other

____

How would you rate the quality of mentoring you received from your parent (US) institution?

- ◯ Very good
- ◯ Moderately good
- ◯ Neutral
- ◯ Moderately bad
- ◯ Very bad

How would you rate the quality of mentoring you received from your LMIC site?

- ◯ Very good
- ◯ Moderately good
- ◯ Neutral
- ◯ Moderately bad
- ◯ Very bad

How supportive would you rate the training environment at your LMIC site?

- ◯ Very supportive
- ◯ Moderately supportive
- ◯ Neutral
- ◯ Moderately unsupportive
- ◯ Very unsupportive

What recommendations do you have for recruiting more global health researchers from historically underrepresented/minority groups? Check all that apply.	☐ More online advertisements about global health research fellowship opportunities<br>☐ More exposure to role models in global health research<br>☐ Increase exposure to global health through personal international travel<br>☐ More exposure to global health research in my classes/coursework<br>☐ Improved financial support for global health research experiences<br>☐ Increased support from my research mentors<br>☐ Increased support for the next steps in my career (gaining faculty positions or career development grants)<br>☐ More technical support for completing the fellowship application online<br>☐ More dissemination of information about global health research fellowships<br>☐ Other (specify and/or elaborate on options checked)
Please specify other	__________________________________
Do you have any additional feedback about your global health research training experience you would like to share from the perspective of historically underrepresented/minority groups ?	__________________________________

E. DEMOGRAPHICS Continued

Thank you very much for completing the survey to this point. To help us better understand the results from the overall survey, we would appreciate very much if you could provide us a little more information about yourself.

What is your gender? (choose one)

- ◯ Male
- ◯ Female
- ◯ Trans man
- ◯ Trans woman
- ◯ Queer/nonconforming neither exclusively male nor female
- ◯ Other (please specify)
- ◯ Prefer not to answer

Please specify other

____

What is the highest degree/ level of training you have obtained?

- ◯ High school or less
- ◯ College (2-year degree)
- ◯ College (4-year degree)
- ◯ Graduate
- ◯ Postgraduate (Fellowship, residency)

Please specify graduate degree

- ☐ MPH
- ☐ PhD
- ☐ MD
- ☐ MBA
- ☐ MSN
- ☐ Other (please specify)

Please specify other

____

What is the highest degree obtained by your father?

- ◯ High school or less
- ◯ College (2-year degree)
- ◯ College (4-year degree)
- ◯ Graduate (MPH, PhD, MD, Other)
- ◯ Postgraduate (Fellowship, residency)
- ◯ Unknown

What is the highest degree obtained by your mother?

- ◯ High school or less
- ◯ College (2-year degree)
- ◯ College (4-year degree)
- ◯ Graduate (MPH, PhD, MD, Other)
- ◯ Postgraduate (Fellowship, residency)
- ◯ Unknown

In which country were you born?

- ◯ United States of America
- ◯ Canada
- ◯ Mexico
- ◯ Nigeria
- ◯ Other (specify)
- ◯ Do not know

Please specify other

____

In which country was your father born?	◯ United States of America<br>◯ Canada<br>◯ Mexico<br>◯ Nigeria<br>◯ Other (specify)<br>◯ Do not know
Please specify other	__________________________________
In which country was your mother born?	◯ United States of America<br>◯ Canada<br>◯ Mexico<br>◯ Nigeria<br>◯ Other (specify)<br>◯ Do not know
Please specify other	__________________________________

Thank you for your participation! We greatly appreciate your time and energy.
